# Angiosome-Targeted Infrapopliteal Angioplasty: Impact on Clinical Outcomes—An Observational Study

**DOI:** 10.3390/jcm13030883

**Published:** 2024-02-02

**Authors:** Mircea Ionut Popitiu, Vlad Adrian Alexandrescu, Giacomo Clerici, Stefan Ionac, Gloria Gavrila-Ardelean, Miruna Georgiana Ion, Mihai Edmond Ionac

**Affiliations:** 1 Research Center in Vascular and Endovascular Surgery, “Victor Babes” University of Medicine and Pharmacy, 300041 Timisoara, Romania; stefan.ionac@umft.ro (S.I.); gloria.gavrila-ardelean@student.umft.ro (G.G.-A.); ion.miruna.g@gmail.com (M.G.I.); mihai.ionac@gmail.com (M.E.I.); 2 Cardio-Vascular and Thoracic Surgery Department, CHUp Sart-Tilman Hospital, University of Liège, 4000 Liège, Belgium; vlad.alexandrescu@vivalia.be; 3 San Carlo Clinic, 20026 Paderno Dugnano, Italy; gclerici@casacura.it

**Keywords:** angiosome concept, percutaneous transluminal angioplasty, diabetes, critical limb-threatening ischemia, drug-coated balloon

## Abstract

**Background**: Revascularization based on the angiosome concept (AC) is a controversial subject because there is currently no clear evidence of its efficacy, due to the heterogeneity of patients (multiple and diverse risk factors and comorbidities, multiple variations in the affected angiosomes). Choke vessels change the paradigm of the AC, and the presence or absence of the plantar arch directly affects the course of targeted revascularization. The aim of this study was to evaluate the effect of revascularization based on the AC in diabetic patients with chronic limb-threatening ischemia (CLTI). **Methods:** This retrospective analysis included 51 patients (40 men, 11 women), with a mean age of 69 years (66–72) and a total of 51 limbs, who presented with Rutherford 5–6 CLTI, before and after having undergone a drug-coated balloon angioplasty (8 patients) or plain balloon angioplasty (43). Between November 2018 and November 2019, all patients underwent below-the-knee balloon angioplasties and were followed up for an average of 12 months. The alteration of microcirculation was compared between directly and indirectly revascularized angiosomes. The study assessed clinical findings and patient outcomes, with follow-up investigations, comparing wound healing rates between the different revascularization methods. Patient records and periprocedural leg digital subtraction angiographies (DSA) were analyzed. Differences in outcomes after direct revascularization and indirect percutaneous transluminal angioplasty (PTa) were examined using Cox proportional hazards analysis, with the following endpoints: ulcer healing, limb salvage, and also amputation-free survival. **Results:** Direct blood flow to the angiosome supplying the ulcer area was achieved in 38 legs, in contrast to 13 legs with indirect revascularization. Among the cases, there were 39 lesions in the anterior tibial artery (ATA), 42 lesions in the posterior tibial artery (PTA), and 8 lesions in the peroneal artery (PA). According to a Cox proportional hazards analysis, having fewer than three (<3) affected angiosomes (HR 0.49, 95% CI 0.19–1.25, *p* = 0.136) was associated with improved wound healing. Conversely, wound healing outcomes were least favorable after indirect angioplasty (*p* = 0.206). When adjusting the Cox proportional hazard analysis for the number of affected angiosomes, it was found that direct drug-coated angioplasty resulted in the most favorable wound healing (*p* = 0.091). At the 1-year follow-up, the major amputation rate was 17.7%, and, according to a Cox proportional hazards analysis, atrial fibrillation (HR 0.85, 95% CI 0.42–1.69, *p* = 0.637), hemodialysis (HR 1.26, 95% CI 0.39–4.04, *p* = 0.699), and number of affected angiosomes > 3 (HR 0.94, 95% CI 0.63–1.39, *p* = 0.748) were significantly associated with poor leg salvage. Additionally, direct endovascular revascularization was associated with a lower rate of major amputation compared to indirect angioplasty (HR 1.09, 95% CI 0.34–3.50, *p* = 0.884). **Conclusions:** Observing the angiosomes concept in decision-making appears to result in improved rates of arterial ulcer healing and leg salvage, particularly in targeted drug-coated balloon angioplasty for diabetic critical limb ischemia, where multiple angiosomes are typically affected.

## 1. Introduction

Over the past 15 years, the scientific literature has explored the notion that revascularization through endovascular or surgical bypass, guided by the AC, could provide valuable insights into the vascular mapping of the foot when treating individuals diagnosed with CLTI [1,2,3,4,5].

### 1.1. Chronic Limb-Threatening Ischemia

The Global Vascular Guidelines (GVG) recommend using the term CLTI instead of “critical” or “severe” limb ischemia. This preference arises because the latter terms designate specific hemodynamic thresholds [2,5], thereby overlooking the broader spectrum and interconnection of components beyond ischemia [2,6,7]. These components play a partial role in the high rate of major amputations and the low rate of long-term survival [2,5,8,9].

CLTI symptoms include peripheral artery disease associated with rest pain with or without skin lesions and a diabetic foot ulcer (DFU) that persists for more than 2 weeks [10].

This condition stands as a primary cause of hospitalization, lower limb amputation, and diminished health-related quality of life (HRQL), leading to substantial treatment costs for those afflicted [11]. Additionally, it poses a significant risk of cardiovascular complications and carries a markedly high mortality rate [12,13]. With major amputation rates surpassing 30%, and mortality at 1 year occurring in approximately 25% of the cases [14], critical limb ischemia is characterized by low awareness, insufficient early-stage diagnosis, and considerable variability in medical practice patterns [5], resulting in a wide range of treatment approaches and clinical outcomes [2,5,15,16,17].

### 1.2. Diabetes Mellitus

A significant risk factor in the management of critical limb ischemia is type 2 diabetes [18]. The literature provides various statistics on the risks associated with diabetic patients: one in four patients ultimately develops an ulcer; in 85% of major amputations in diabetic patients, there is a preexisting ulcer; 65% of ulcers exhibit an ischemic component [19], and peripheral arterial disease is present in 10% to 40% of patients diagnosed with diabetes [20].

### 1.3. WIfI Classification

The Society for Vascular Surgery has established a classification system for accurate diagnosis of the lower extremity risk stage for the management of CLTI [21]. This system relies on three factors: wound, ischemia, and foot infection (WIfI), which elevate the likelihood of amputation and contribute to clinical management [4,22,23,24]. As wound complexity and infection severity increase, revascularization also becomes increasingly necessary [1,25].

### 1.4. Angiosomes Concept

The year 1987 stands as a chronological milestone for the AC when Taylor and Palmer defined it by expanding upon the literature previously published by anatomists in the field of reconstructive surgery [1,4,26]. They divided the body into three-dimensional anatomical tissue territories, each perfused and drained by specific named source arteries [27]. Taylor and Palmer demonstrated that the arteries supplying these tissue areas provide blood supply to the skin, subcutaneous tissue, fascia, muscle, and bone [28]. They named these composite units “angiosomes” and concluded that the thought process behind incisions and flaps should be based on the arterial map that they have described [29]. 

The distribution of angiosomes was demonstrated in 2006 when Attinger et al. injected differently colored methyl methacrylate solutions in the arteries of the lower legs of 50 dissected cadavers [1,4,30,31]. They described six angiosomes with an origin in three principal arteries, featuring multiple arterial–arterial connections. Their research revealed the presence of three angiosomes on the plantar foot (supplied by the posterior tibial artery), two on the ankle and rear foot (peroneal artery), and one on the dorsum of the foot (anterior tibial artery) [1,32,33].

Choke vessels border each angiosome [34], providing indirect interconnection between multiple ones [35].

## 2. Materials and Methods

### 2.1. Study Design and Ethical Approval

A retrospective observational study was conducted on a database of patients who underwent endovascular revascularization based on the angiosomes concept at the Department of Vascular Surgery in a public academic hospital. This research was designed based on the Strengthening the Reporting of Observational Studies in Epidemiology (STROBE) Statement of cohort studies. The research was conducted in accordance with the principles of the Declaration of Helsinki. The requirement for informed consent was waived because the patients’ data were retrospectively and anonymously evaluated. The practicians declare that all procedures followed medical protocols. The research was evaluated and approved by the Ethical Committee of “Pius Brinzeu” Public Hospital Timisoara, Romania (Approval no. 147/26 October 2018) and by the Ethical Committee of “Victor Babes” University of Medicine and Pharmacy Timisoara, Romania (approval no. 28/28 September 2018).

### 2.2. Inclusion and Exclusion Criteria

The following table outlines the criteria for patient inclusion and exclusion in the study. (Table 1). 

### 2.3. Definitions

Following the AC, a target artery is an infrapopliteal vessel responsible for directly supplying the diabetic foot wound with blood, while a non-target artery achieves this indirectly, through collateral circulation.

The definition of critical limb ischemia includes resting pain lasting more than two weeks, an ankle systolic pressure below 50 mmHg or an ankle pressure level that cannot be measured, along with the presence of ulceration, gangrene, or non-healing foot wounds. 

Defined by its extent related to the ankle joint, a minor amputation is performed distally, while a major amputation occurs above the ankle joint.

The survival rate with no major amputation around the knee joint, throughout the follow-up, represents the rate of leg salvage. The present study assesses the leg salvage rate over the initial 12 months.

We consider a wound healed when the tissue defect is fully epithelized through secondary intention or following any supplementary local surgery targeting the ulcer. A wound that persists in an open state after the follow-up is deemed unhealed

### 2.4. Patients and Variables

51 patients with CLTI were selected, comprising 40 men (76.5%) and 11 women (23.5%), who underwent revascularization based on the angiosomes concept (28 direct, 12 indirect, and 11 received both approaches). Among the patients, 43 were treated with a commercial plain balloon, while the remaining 8 were treated with a commercial drug-eluting balloon. The study cohort included only patients classified as Rutherford category 5 to 6. Data were retrospectively collected from 1 November 2018 to 1 November 2019. The research, along with its point follow-up investigations, concluded on 1 November 2020. The average follow-up period was 12 months, but 4 patients were lost to follow-up as they did not participate in the follow-up investigation. 

Patients with false values of the ankle–brachial index (ABI > 1.3) were excluded from the calculation. Dorsal and plantar angiosomes of the foot were evaluated pre and post intervention by duplex ultrasound imaging and digital substraction angiography.

### 2.5. Outcomes of Interest and Follow-Up Protocols

Clinical aspects of the wound were recorded during the follow-up evaluation and documented through photography. ABI and duplex ultrasound were used to evaluate revascularization permeability. The wounds were classified at the time of the first investigation using the wound, ischemia, and foot infection (WIfI) scores from the Society of Vascular Surgery.

### 2.6. Statistical Analyses

All statistical analyses were performed using the standard software package IBM SPSS v. 27 from SPSS Inc. (Chicago, IL, USA), and the licensed version 1808 of Microsoft Excel 2019 (Microsoft, WA, USA). Data normality was assessed using the Kolmogorov-Smirnov test, and they are presented as categorical variables or frequency distributions. Mean values +/− S.D. were used for the variables with a Gaussian distribution; Bravais-Pearson or Spearman’s rank correlation coefficient with the following strength ranges were used for linear monotonic relationships: weak (between 0.02–0.39), moderate (between 0.40–0.69), and strong (0.70–1.00). A two-way ANOVA followed by Bonferroni correction was used to identify the difference between more than two groups; the significance was set based on a *p* < 0.05.

## 3. Results

Studying the two ways of revascularization used the following parameters as endpoints, which have been evaluated for 12 months after the clinical intervention: leg salvage, amputation-free survival, and wound healing.

Table 2 presents the patients’ comorbidities and offers a simplified representation of the main characteristics of patients, categorized into two groups by the type of angioplasty (direct vs. indirect).

Table 3 presents a comparative analysis of the targeted arteries within the two studied groups. Table 3 presents a series of correlations between the variables that were studied in this research.

The main arteries targeted were ATA (n = 25), PTA (n = 11), Per (n = 13), and ATA + PTA (n = 8). 

The following direct correlations were found: strong (leg salvage at 12 months vs. survival at 12 months, and healing at 12 months vs. leg salvage at 12 months), moderate (hemodialysis vs. renal failure, angiosome no. vs. wound PTA, and angiosome no. vs. wound ATA), and weak (diabetes insulin vs. sex, and angiosome no. vs. leg salvage at 12 months). Indirect correlations were also observed: strong (death vs. leg salvage at 12 months, and death vs. healing at 12 months), moderate (wound per vs. wound ATA, and minor amputation vs. ABI pre), and weak (dyslipidemia vs. hemodialysis, and plagues on both legs vs. diabetes oral ad) (Table 4).

According to a Cox proportional hazards analysis, having fewer than three (<3) affected angiosomes (HR 0.49, 95% CI 0.19–1.25, *p* = 0.136) was associated with improved wound healing.

On the other hand, the following figures comparatively present various evolutions. 

**Figure 1:**
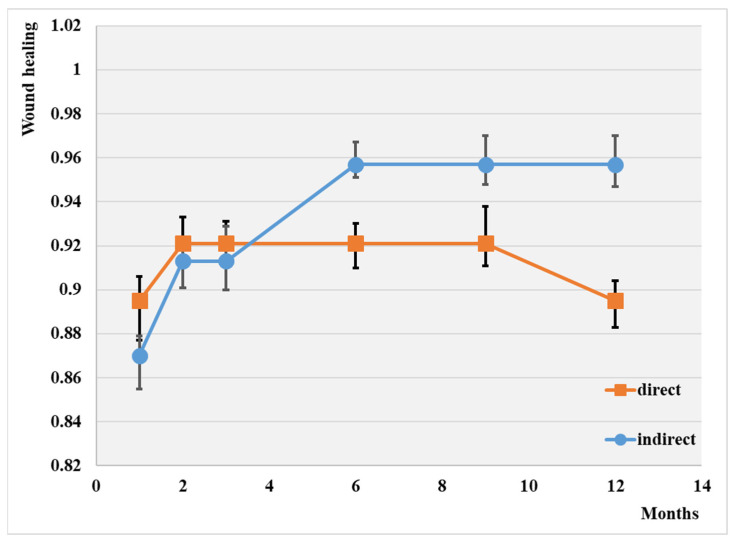
Wound healing depending on the type of angioplasty; *p* < 0.01.

Thus, Figure 1 illustrates wound healing depending on the type of angioplasty in our studied groups.

The overall wound healing rate at 1 year was 90.2%. Note that the highest rate of wound healing was attained through indirect revascularization (95.7%), and the lowest was with simple balloons (89.5%). It appears that the indirect procedure has a more efficient influence on wound healing, although the difference between the two groups is not substantial. 

The highest wound healing rate at one year was attained following direct drug-coated balloon angioplasty (69%), while the lowest was observed after indirect plain balloon revascularization (35%).

**Figure 2:**
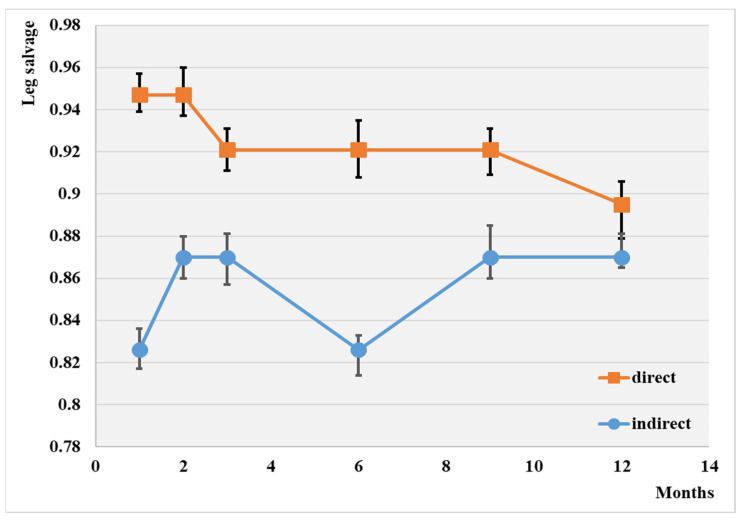
Evolution of leg salvage depending on the type of angioplasty; *p* < 0.05.

The endovascular procedure used for leg salvage is displayed comparatively in Figure 2.

The leg salvage rate at the 1-year mark was 88.3% overall. Notably, the highest rate of leg salvage (89.5%), was observed with direct revascularization, while the lowest was observed in the case of the indirect procedure (87.0%). No significant difference was observed between the two groups at the end of this investigation. 

**Figure 3:**
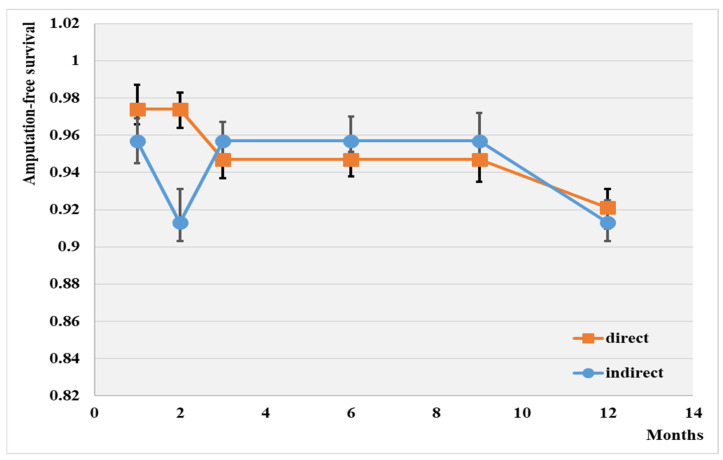
Amputation-free survival depending on the type of angioplasty; *p* < 0.01.

The overall 1-year major amputation-free survival rate was 91.8% (Figure 3).

The highest wound healing rate was achieved with the direct intervention (92.1%), while the lowest was observed with the indirect method (91.3%). No significant difference was observed between the two groups after 12 months of this investigation.

**Figure 4:**
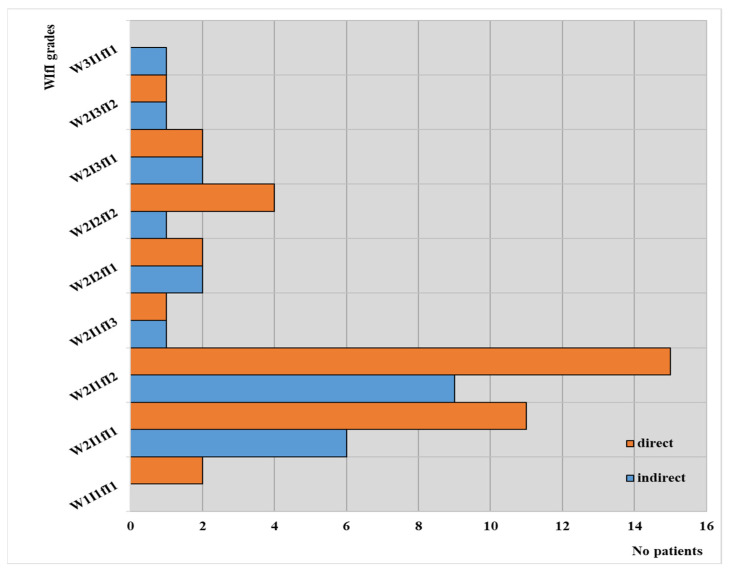
WIfI classification according to the type of angioplasty in our observed groups.

WIfI grades according to the type of angioplasty in our observed groups are depicted in Figure 4.

These results can support a correlation between patients treated by direct revascularization and the WIfI grades; in their cases, the highest value (3) was not found for any of the three parameters, with most of these patients falling between W2I1fI1 and W2I1fI2. On the other hand, it is worth mentioning that no patients from the group with indirect intervention achieved the best value for WIfI grades. 

**Figure 5:**
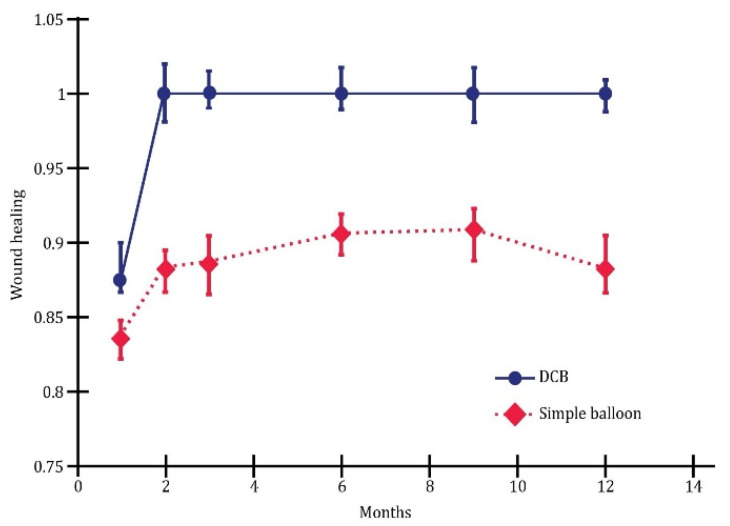
Wound healing; *p* < 0.05.

**Figure 6:**
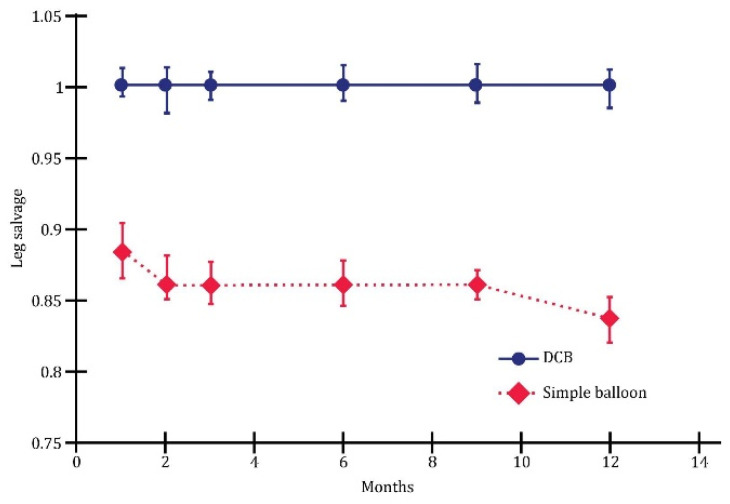
Leg salvage; *p* < 0.01.

**Figure 7:**
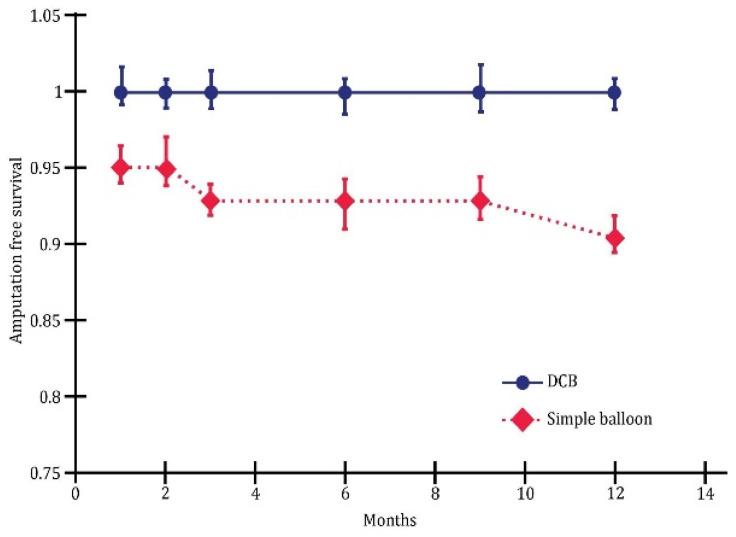
Survival without amputation; *p* < 0.05.

The multivariate analysis revealed substantial differences between plain balloon angioplasty and drug-coated balloon (DCB) in endovascular revascularization. Overall, this study demonstrated that DCB outperformed plain balloons in terms of one-year wound healing (Figure 5), limb salvage (Figure 6), and the rate of survival without amputation (Figure 7).

## 4. Clinical Cases

### 4.1. Case 1—Direct Revascularization-Minor Amputation

**Figure 8:**
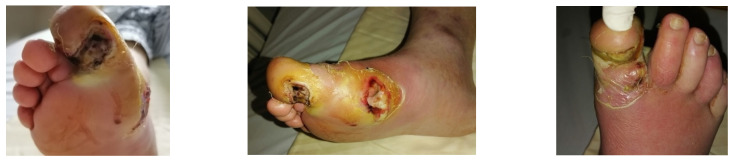
Clinical aspect.

**Figure 9:**
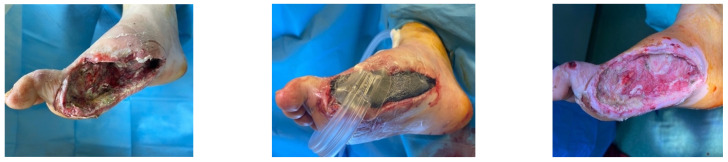
Hallux amputation, excisional debridement, vacuum-assisted closure (VAC) placement.

**Figure 10:**
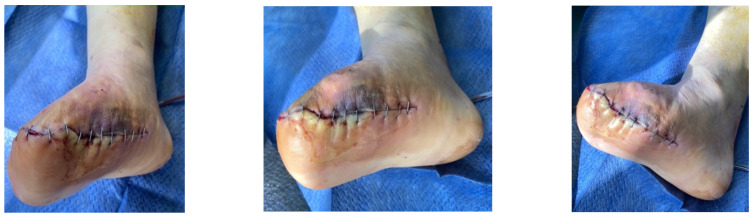
Unfavorable evolution post-transmetatarsal amputation.

**Figure 11:**
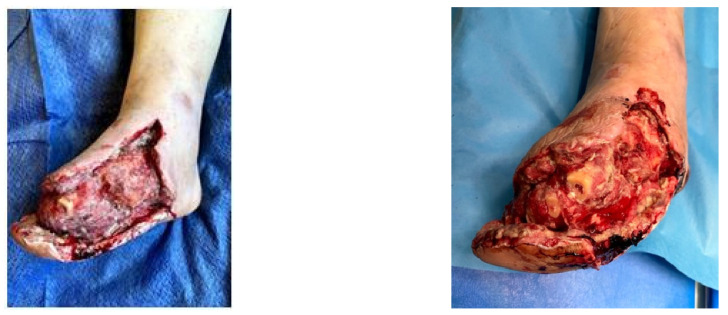
Aspect after excisional debridement and VAC therapy.

**Figure 12:**
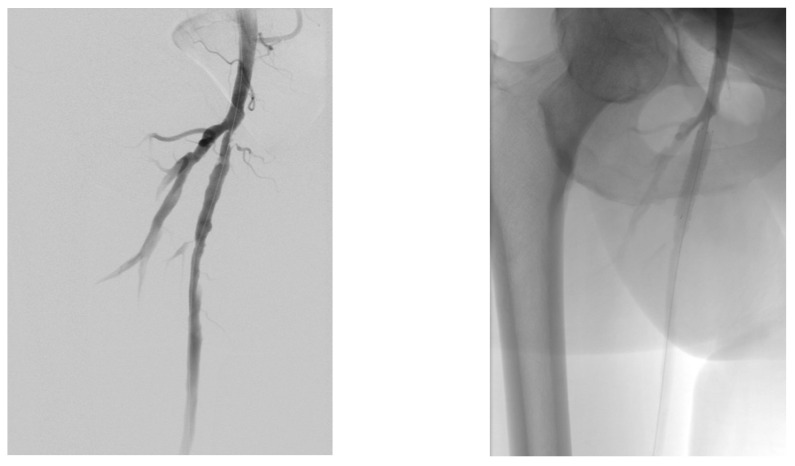
Angioplasty with SFA stent at the origin.

**Figure 13:**
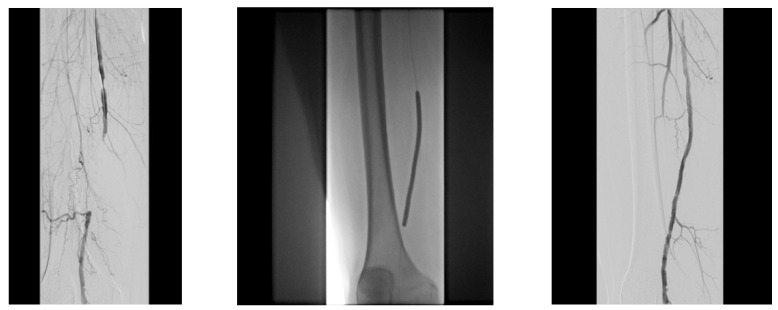
Pre and post SFA balloon PTa.

**Figure 14:**
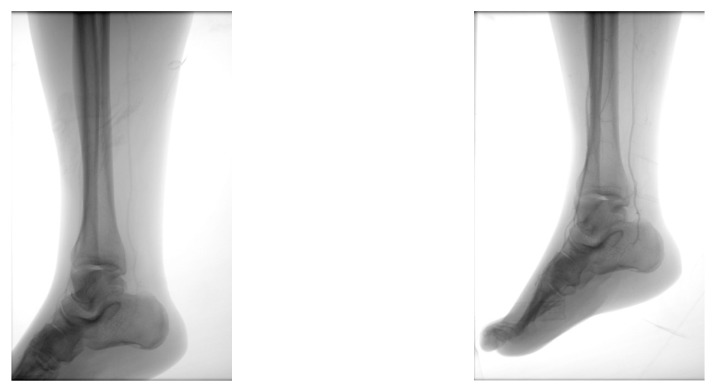
ATA balloon PTa.

**Figure 15:**
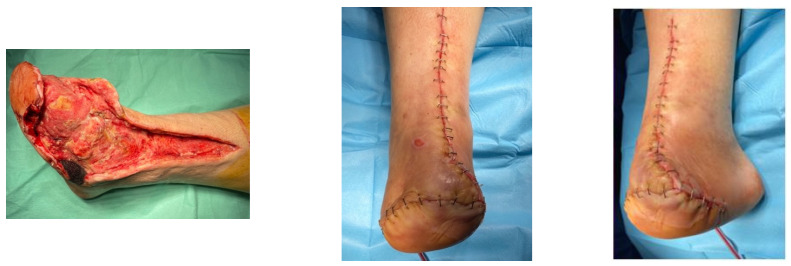
Secondary suture post-Chopart amputation.

**Figure 16:**
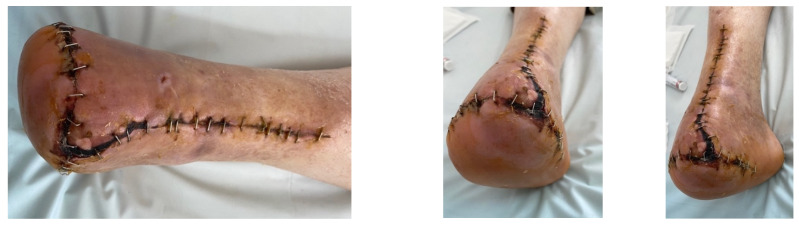
Follow-up at 2 weeks.

**Figure 17:**
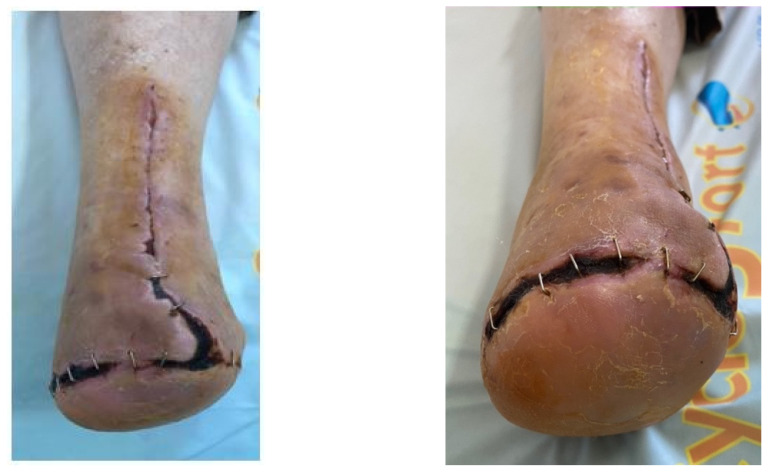
Follow-up at 3 weeks.

**Figure 18:**
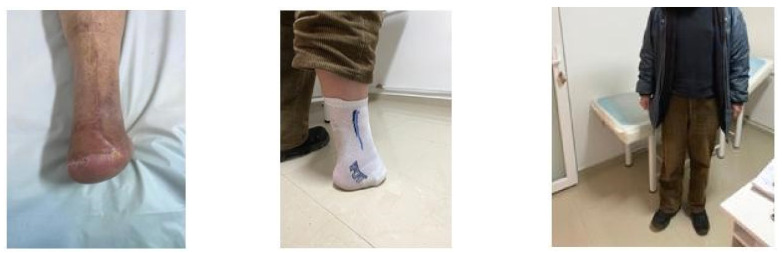
Follow-up at 3 months.

**Figure 19:**
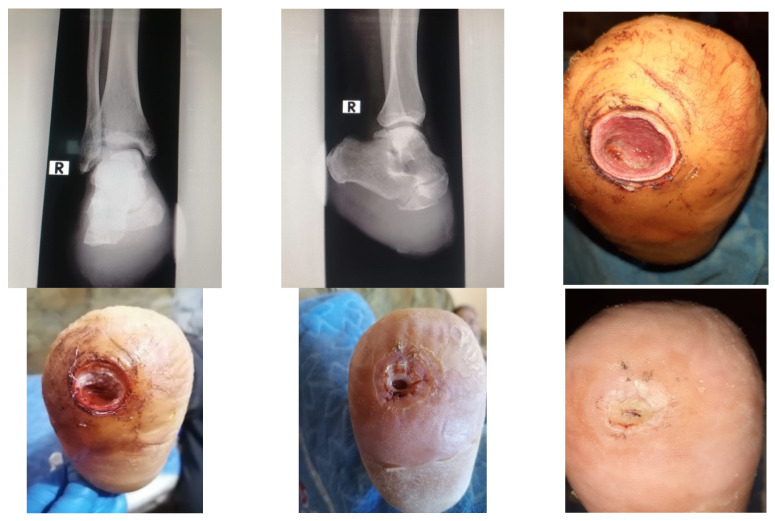
1-year Follow-up.

A diabetic patient presents with a lesion in the medial plantar angiosome—PTA and dorsal angiosome—ATA (Figure 8). After the unsuccessful attempt of transmetatarsal amputation (Figure 9, Figure 10 and Figure 11), diagnostic angiography is performed, PTA with a superficial femoral artery (SFA) stent in the proximal 1/3, PTA with an SFA balloon in the middle 1/3, and PTA with an ATA balloon—direct revascularization (Figure 12, Figure 13 and Figure 14). A Chopart amputation is carried out with good evolution (Figure 15). A good evolution was observed at the 2 week follow-up, (Figure 16), at the 3 week follow-up (Figure 17), and the 3 month follow-up (Figure 18). At the 1-year follow-up, the patient presents a superinfected pressure ulcer without bone involvement, which granulates after excisional debridement, antibiotic treatment according to antibiogram, and wet dressings (Figure 19). Healing is achieved in about one month.

### 4.2. Case 2—Indirect Revascularization-Major Amputation

**Figure 20:**
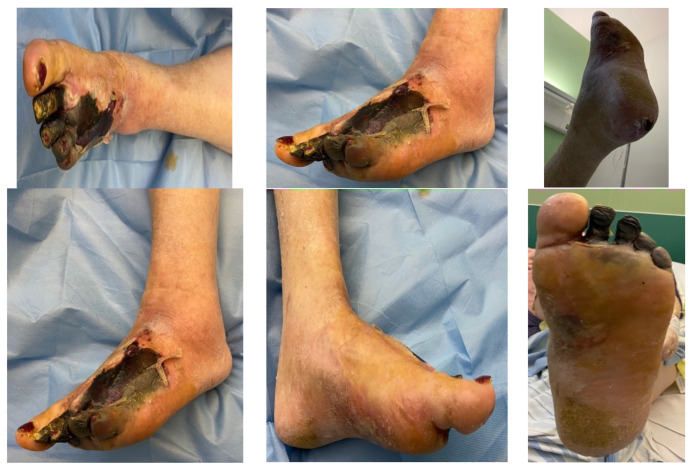
Clinical aspect on admission; Affected angiosomes—dorsal angiosome—ATA and lateral calcaneal angiosome—Peroneal artery.

**Figure 21:**
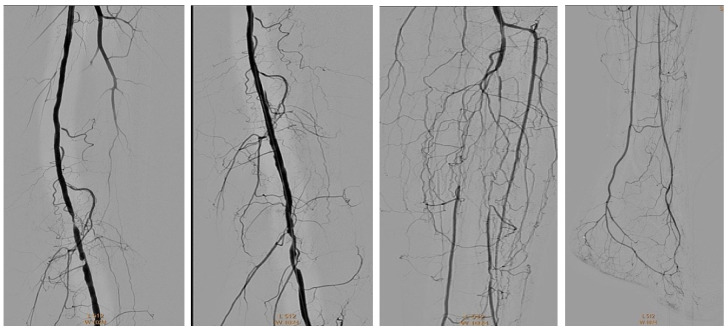
Pre and post SFA balloon PTa.

**Figure 22:**
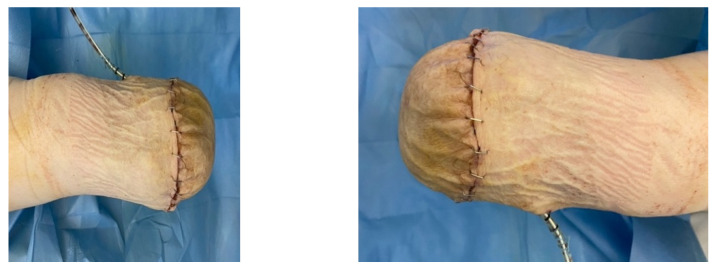
Below-the-knee amputation.

**Figure 23:**
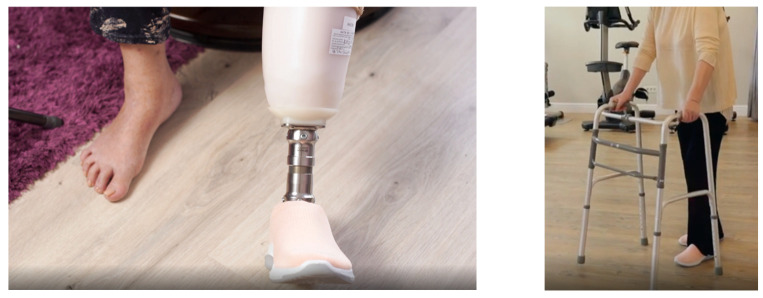
1 year Follow-up.

A female patient with diabetes mellitus and critical ischemia presented with wet gangrene affecting the second to fifth toes of the left foot, along with dry necrosis on the dorsal aspect of the forefoot (ATA) and wet gangrene in the lateral calcaneal region (A per) (Figure 20). The management included amputation of toes II–V and excisional debridement of the dorsal aspect of the left forefoot, coupled with the evacuation of a plantar abscess. Despite SFA balloon angioplasty for indirect revascularization, the patient’s condition showed unfavorable progression, necessitating a below-the-knee amputation (Figure 21 and Figure 22). An alternative approach could have involved ATA or PTA balloon PTa for direct revascularization, potentially enabling healing in the context of a Chopart amputation. The 1-year follow-up revealed promising results, as the patient was protheticaly restord and has resumed normal activity (Figure 23).

## 5. Discussion

### 5.1. Direct vs. Indirect Revascularization

According to the GVG, there is ample controversy surrounding the benefits of performing angiosome-guided revascularization for multiple reasons [36]. Firstly, assigning foot wounds to a specific angiosome without any ambiguity is limited to a small percentage of cases. This challenge is particularly notable since lesions affecting the toes, supplied by both the anterior tibial and posterior tibial arteries (ATA and PTA), constitute more than half of all cases [37]. Secondly, a technical consideration arises concerning the availability of the target artery for the specified angiosome [38]. Additionally, the discussion extends to the comparative hemodynamic and clinical effectiveness of both “direct” and “indirect” revascularization methods [39].

Direct revascularization involves restoring blood supply directly to an angiosome that includes an ulcer, whereas indirect revascularization aims to restore blood supply to a neighboring angiosome that does not contain an ulcer [40]. The published literature mostly focuses on retrospective evaluations of the concept concerning the following targets: wound healing and amputation-free survival. Nevertheless, these objectives are influenced by numerous other factors beyond the scope of direct and indirect revascularization [2,41,42,43,44,45].

Wounds are different, leading to distinct approaches and varying blood flow requirements for healing. The effectiveness of revascularization, particularly through a direct endovascular approach, is contingent upon the specific stage of the wound [1,46].

In their review of foot angiosomes, Ferraresi et al. recommend conducting comprehensive and qualitative imaging studies of the vascular network in the leg and foot before deciding on a revascularization approach. This is to establish the distribution of the main arteries and their spatial relations, enabling a tailored consideration of the patient’s individual anatomy [1,47].

As mentioned earlier, the main target of revascularization in individuals with CLTI is to promote wound healing and ensure survival without amputation [48,49].

In terms of revascularization strategies, there are multiple approaches available [1,50,51].

### 5.2. Endovascular versus Bypass

In their study of patients with CLTI who have received distal bypasses, Azuma et al. discovered similar healing rates between the targeted and non-targeted angioplasty groups, after reducing background differences. This was achieved using propensity score methods [1,51]. Their deduction suggests that, in the context of bypass surgery for patients with non-end-stage renal disease, the angiosome concept seems to lack significant importance. They emphasized that factors related to ischemic wounds, such as localization and extent, along with comorbidities, play a more substantial role in influencing wound healing outcomes [1,52].

In surgical revascularization, whether employing a direct or indirect approach, the selection of the most appropriate vessel for revascularization is typically necessary. On the other hand, endovascular revascularization allows for the precise targeting of the specific angiosome, which is an important advantage [53].

However, this approach calls for prolonged and intricate techniques, which, regrettably, do not always yield favorable outcomes [54]. Bypass surgery remains crucial due to its ability to generate increased local pressure and maintain physiological pulsatile flow. Additionally, endovascular techniques are also performed to enable multiple reconstructions of vessels below the knee and ankle [55]. Endovascular therapy presents the possibility of opening multiple (>1) tibial vessels [56,57,58,59,60,61].

Settembre et al. conducted a study involving 580 patients who received different treatments for CLTI associated with foot ulcer or gangrene. Among these participants, 407 underwent endovascular revascularization, while 173 had surgical revascularization targeting the infrapopliteal arteries. The findings showed that angiosome-targeted revascularization is important due to endovascular thrombectomy (EVT), particularly in the absence of a complete pedal arch [62]. The study demonstrated that, in addition to elevated C-reactive protein (CRP), rheumatoid arthritis, and the total number of affected angiosomes, a factor that independently predicts poor leg salvage is an incomplete pedal arch identified during pre-surgery angiography [48,62].

In their meta-analysis, Chae and Shin discovered that angiosome-targeted angioplasty significantly improved the overall rate of limb salvage, and wound healing [63]. 

It is imperative to acknowledge that the treatment of CLTI cannot solely rely on surgery, even if successful. Postoperative care, encompassing medication and wound management, is of utmost importance. These aspects are invaluable, particularly in patients with underlying pathologies such as end-stage renal disease (ESRD) or diabetes. The reason is that in such cases, wound healing strength is diminished, and the immune system is compromised.

## 6. Limitations

The conclusions of our research should be observed with some limitations. Our study is an observational study conducted on a relatively small sample size of 51 patients. This is because it resulted from analyzing the activity of a single center. Not all patients from our clinic benefit from endovascular techniques. Predominantly, open surgery is practiced, utilizing vein bypass. The study lacks a control group for comparison; the only aspect used was the comparison between the group that received direct revascularization according to the AC and the group with indirect revascularization according to the AC. This is because our center has already implemented the working protocol according to the AC, and there are no patients endovascularly revascularized without considering anatomical aspects of the AC. Also, we did not have access to the genetic information of the patients.

## 7. Conclusions

In CLTI cases, direct endovascular revascularization results in markedly superior rates of wound healing and leg salvage when compared to indirect revascularization. The AC is feasible for most patients treated with an endovascular approach, although only 27.45% of the tissue lesions are exclusively localized to a single angiosome. When a wound extends across more than one angiosomes in the heel or forefoot region, targeting all affected angiosomes may lead to better clinical outcomes.

The factors linked to prolonged wound healing times include the involvement of more than three (>3) affected angiosomes and indirect percutaneous transluminal angioplasty (PTa). Therefore, integrating the AC into decision-making appears to result in improved leg salvage rates and wound healing, especially with direct PTa. Our study underscores the significant role of the AC in endovascular treatment, emphasizing that indirect revascularization is associated with the least satisfactory clinical outcomes.

## 8. The Angiosome Concept in Clinical Practice

Clinical practice should take the following into consideration:

(1) Identification of the affected angiosomes and patent arteries of the foot and establishment of the practical, not ideal, technical approach to revascularization.
(2) Precise imaging diagnosis of the plantar arch and Choke vessels and identification of the vascular distribution particularities of the affected foot. The presence of Choke vessels represents a key decision factor for the necessity of direct endovascular revascularization but does not influence indirect surgical revascularization.
(3) Placing importance on the presence of Choke vessels in patients with CLTI (critical limb-threatening ischemia) when making decisions about targeted revascularization based on the AC.
(4) Clinical classification of the degree of wound depth is a key aspect of targeted revascularization based on the AC.
(5) The AC should be taken into consideration for endovascular revascularization and bypass procedures when feasible, as it is directly linked with the clinical success of the procedures.
(6) In the case of severe wounds, attempts should be made for multi-vascular endovascular revascularization, both direct and indirect.

## Figures and Tables

**Table 1:** Inclusion and Exclusion criteria.

Inclusion Criteria	Exclusion Criteria
1. Patients with Rutherford Stage 5–6 or Leriche-Fontaine IV.	1. Allergy to iodine.
2. Diabetic patients with syndromes of “Neuro-ischemic diabetic legs”.	2. Liver failure.
3. Patients requiring infragenicular revascularization via the endovascular technique.	3. Pregnancy
4. The presence of arteries viable for revascularization.	4. Hyperthyroidism.
5. The number of angiosomes affected by the wound >1.	5. Pulmonary arterial hypertension.
6. Comorbidities.	6. End-stage renal failure and dialysis.
7. Critical limb-threatening ischemia.	7. Acute ischemia.
8. Complications of the underlying disease.	8. Fem-popliteal aneurysmal disease.
9. Smoking.	9. Extensive gangrene of the leg with systemic sepsis and inevitable amputation.
10. Age > 50 years.	10. Myocardial infarction less than three months ago.
11. ABI < 0.7.	11. Anterior and thrombosed infra-genicular bypass.
12. Consent for surgery.	12. Chronic treatment with cortisone or cytostatics.
13. Rest pain	13. Dementia or psychotic behavior.
14. Patients with endovascular revascularization solution.	14. Total absence of collaterals of the foot (“Desert foot”).
15. Essential arterial hypertension.	15. Diagnosed thrombophilia.

ABI = Ankle–Brachial Index.

**Table 2:** Comparative patients’ comorbidities and characteristics.

Characteristics	Angioplasty Type		*p*
	Direct	Indirect	
Age, years	68.4 ± 8.7	67.9 ± 6.3	0.696
Age ≥ 80 years	4 (10.5)	0 (0.0)	0.041
Female	9 (23.7)	5 (21.7)	0.781
Smoking	13 (34.2)	9 (39.1)	0.413
Diabetes Oral Ad	26 (68.4)	16 (69.6)	0.756
Insulin	16 (42.1)	10 (43.5)	0.809
Hypertension	35 (92.1)	22 (95.7)	0.672
Renal failure	8 (21.1)	5 (21.7)	0.693
Hemodialysis	3 (7.9)	1 (4.4)	0.129
Stroke	5 (13.2)	6 (26.1)	0.140
Dyslipidemia	31 (81.6)	19 (82.6)	0.527
Coronary heart disease	6 (15.8)	5 (21.7)	0.092
Heart disease	21 (55.3)	14 (60.9)	0.483
Heart failure	18 (47.4)	12 (52.2)	0.371
Atrial fibrillation	6 (15.8)	7 (30.4)	0.529
Myocardial infarction	4 (10.5)	1 (4.4)	0.453
ABI pre-	0.40 ± 0.18	0.42 ± 0.25	0.374
post-	0.75 ± 0.33	0.68 ± 0.37	0.392
Angiosome no	2.05 ± 0.70	1.87 ± 0.76	0.881

**Table 3:** Comparative data of patients.

Targeted Atery	Angioplasty Type		*p*
	Direct	Indirect	
PTA ATA	25 (65.8)	12 (52.2)	0.588
PTA	11 (28.9)	7 (30.4)	0.704
Truncus	8 (21.1)	11 (47.8)	0.147
Per	13 (34.2)	10 (43.5)	0.467

ATA = anterior tibial artery; PTA = posterior tibial artery; Per = peroneal artery.

**Table 4:** Correlation coefficients of studied variables.

Correlation	Angioplasty Type	
	Direct	Indirect
Angiosome no. vs. Leg salvage at 12 months	0.026 ^#^	0.282 ^#^
Diabetes insulin vs. Sex	0.403 *	0.388 ^#^
Hemodialysis vs. Renal failure	0.567 **	0.405 ^#^
Dyslipidemia vs. Hemodialysis	−0.364 *	−0.465 *
Minor amputation vs. ABI pre	−0.335 *	−0.502 *
Wounds on both legs vs. Diabetes Oral Ad	−0.347 *	−0.467 *
Wound per vs. Wound ATA	−0.544 **	−0.707 **
Angiosome no. vs. Wound ATA	0.449 **	0.621 **
Angiosome no. vs. Wound PTA	0.585 **	0.640 **
Healing at 12 months vs. Leg salvage at 12 months	0.721 **	0.550 **
Leg salvage at 12 months vs. Survival at 12 months	0.854 **	0.549 **
Death vs. Healing at 12 months	−0.854 **	−1.000 **
Death vs. Leg salvage at 12 months	−1.000 **	−1.000 **

^#^ = non-significant; * = significance at the 0.05 and ** = significance at the 0.01 level (2-tailed).

## Data Availability

No new data were created or analyzed in this study. Data sharing is not applicable to this article.

## Abbreviations

ABI	Ankle-Brachial Index
AC	Angiosome Concept
ATA	Anterior Tibial Artery
CLI	Critical Limb Ischemia
CLTI	Chronic Limb-Threatening Ischemia
DR	Direct Revascularization
DCB	Drug-Coated Balloon
DFU	Diabetic Foot Ulcer
DSA	Digital Subtraction Angiography
ESRD	End-Stage Renal Disease
EVT	Endovascular Thrombectomy
GVG	Global Vascular Guidelines
HRQL	Health-Related Quality of Life
ID	Indirect Revascularization
PAOD	Peripheral Arterial Occlusive Disease
PTA	Posterior Tibial Artery
PTa	Percutaneous Transluminal Angioplasty
Per	Peroneal Artery
SFA	Superficial Femoral Artery
VAC	Vacuum-Assisted Closure
WIfI	Wound, Ischemia, and Foot Infection
